# Response rates and durability of chemotherapy among 62 patients with metastatic Merkel cell carcinoma

**DOI:** 10.1002/cam4.815

**Published:** 2016-07-19

**Authors:** Jayasri G. Iyer, Astrid Blom, Ryan Doumani, Christopher Lewis, Erica S. Tarabadkar, Austin Anderson, Christine Ma, Amy Bestick, Upendra Parvathaneni, Shailender Bhatia, Paul Nghiem

**Affiliations:** ^1^ Department of Medicine/DermatologyUniversity of WashingtonSeattleWashington; ^2^ Department of Radiation OncologyUniversity of WashingtonSeattleWashington; ^3^ Department of Medicine/Medical OncologyUniversity of WashingtonSeattleWashington

**Keywords:** Chemotherapy, durability of response, Merkel cell carcinoma, metastatic, neuroendocrine tumor, progression‐free survival, response rate

## Abstract

Cytotoxic chemotherapy is commonly used to treat advanced Merkel cell carcinoma (MCC). However, its efficacy in distant metastatic MCC patients is unclear, in part because most prior reports aggregated these patients with those receiving adjuvant chemotherapy and combined chemoradiation for whom prognosis and outcomes may differ. In this retrospective study, we analyzed detailed records from 62 patients with distant metastatic MCC treated with cytotoxic chemotherapy. Efficacy outcomes including response rate (RR), durability of response (DOR), progression‐free survival (PFS), and overall survival (OS) were evaluated. In this cohort, platinum plus etoposide was the most commonly used first‐line regimen (69%). RR to first‐line chemotherapy was 55% (34/62) with complete responses (CR) in 13% (8/62) and partial responses (PR) in 42% (26/62) while 6% (4/62) had stable disease and 39% (24/62) had progressive disease. Median PFS was 94 days and median OS was 9.5 months from start of chemotherapy. Among responding patients (*n* = 34), median PFS was 168 days and median DOR was 85 days. Among 30 of the 62 patients who received second‐line chemotherapy, RR was 23% (7/30; 1 CR, 6 PR), median PFS was 61 days, and median DOR was 101 days. In summary, first‐line chemotherapy is associated with a high RR in metastatic MCC, but responses are typically not durable, and the median PFS is only 3 months. These results suggest rapid emergence of chemoresistance in MCC tumors, and may serve as a useful comparator for immunotherapies currently being explored for metastatic MCC.

## Introduction

Merkel cell carcinoma (MCC) is an aggressive skin cancer with a 5‐year mortality of ~40% 1. Approximately 80% of MCC cases are casually linked to the Merkel cell polyomavirus (MCPyV) 2. Survival in MCC is strongly linked to the immune system. Specifically, survival is markedly higher in patients who have CD8 lymphocytes infiltrating their tumors and conversely survival is markedly lower among patients who have chronic immunosuppression 3, 4.In two independent cohorts, 48% of MCC patients developed a recurrence and median time from initial diagnosis to recurrence was 9 months 5, 6. Prognosis for metastatic MCC is typically poor, with median survival of 9.6 months from time of diagnosis of distant metastatic MCC 7, and systemic treatment options have mostly been extrapolated from small cell lung cancer (SCLC), with which MCC shares many similarities 8. Specifically, both MCC and SCLC are aggressive and poorly differentiated neuroendocrine cancers with initial response to any of several cytotoxic chemotherapy agents. Platinum agents plus etoposide are commonly used as the initial systemic treatment modality for both MCC and SCLC 9, 10. However, unlike SCLC, no prospective clinical trials of chemotherapy have been conducted for MCC due to inherent challenges of conducting trials in an uncommon malignancy.

The existing literature mostly comprises retrospective case reports, series, and reviews regarding the efficacy of chemotherapy for MCC. Prior reports are challenging to interpret because they often included responses for combined treatments including chemoradiation, and for patients receiving adjuvant chemotherapy. Moreover, they combine response data for local–regional and distant metastatic disease, which have different prognoses. Based on the current literature, it is therefore difficult to determine the therapeutic efficacy of chemotherapy alone in metastatic MCC. While initial responses appear to be common in metastatic MCC 11, 12, there are few data to assess the durability of these responses. Furthermore, data on progression‐free survival (PFS) is not available. To address these limitations, we carried out this retrospective study of 62 patients on which we had detailed medical records to evaluate efficacy outcomes from cytotoxic chemotherapy, including response rates, durability of response (DOR), and PFS for distant metastatic MCC.

## Methods

All studies were performed in accordance with Helsinki principles and were approved by the Institutional Review Board at the Fred Hutchinson Cancer Research Center (IRB # 6585). All patients included in this study had provided informed consent for enrollment in this IRB‐approved database.

### Inclusion criteria for study cohort

The study cohort included 62 patients with distant metastatic MCC, who had received cytotoxic chemotherapy as initial treatment for metastatic disease, and had adequate clinical and follow‐up information available to allow evaluation of antitumor efficacy outcomes from chemotherapy. These patients were treated at several different institutions, including the University of Washington. Cases were only included if there was adequate information available on the details of chemotherapy including agent(s) used, the dates of administration, and tumor responses (including follow‐up radiologic evaluation). Patients were excluded if tumors could not be evaluated for the efficacy of chemotherapy alone, for example when distant metastatic tumors had been treated with concurrent radiation and/or surgery. Patients were also excluded if they only had an isolated skin MCC lesion distant from the primary MCC, because it is possible that such a lesion could represent a second primary 13 rather than true metastatic disease.

### Data collection

In this retrospective study, we performed a thorough chart review of patients enrolled in the Seattle‐based repository. Patient and tumor characteristics were collected until last follow‐up, death of the patient, or cut‐off date for data collection for this study, 7 January 2014. We collected data on age, sex, number, size, and location of treated metastatic lesions, immune status, exposure to previous and subsequent therapies, lesions targeted or treated using other modalities such as surgery/radiation therapy, treatment dates, scan/physician reports, response to treatment per RECIST (Response Evaluation Criteria In Solid Tumors 1.1 criteria) 14 when available, and acute and late toxicity. Imaging data following first‐line chemotherapy were available for 58 of the 62 patients. In the remaining four cases, we relied on the physician's note to assess the extent of response. Of these four patients, three had cutaneous disease evaluable by physical examination and one patient died of progressive MCC within 2 weeks from the start of chemotherapy.

### Study endpoints and statistical analysis

Objective tumor responses were classified per RECIST 1.1 as complete response (CR), partial response (PR), stable disease (SD), or progressive disease (PD) 14. Efficacy endpoints included response rate (RR) (the number of patients with the best response of CR or PR across all time points divided by the total number of patients receiving therapy), DOR (time from best overall response of partial or complete response to first documented disease progression), PFS (time from treatment initiation to first documented disease progression or death due to any cause), and overall survival (OS) (time from treatment initiation to death due to any cause). Patients without an event were censored at the time of the last tumor assessment of nonprogressive disease or, for survival, the date they were last known to be alive. Survival analyses were carried out using the Kaplan–Meier method 15.

## Results

### Patient and tumor characteristics

Sixty‐two patients (47 men and 15 women) diagnosed between 2002 and 2014 with distant metastatic MCC, and who met the key eligibility criteria were included in this study (Table**** 1). Fourteen (23%) of the 62 patients had conditions associated with compromised immune function including CLL (*n* = 4), non‐Hodgkin's lymphoma (*n* = 1), mycosis fungoides (*n* = 1), HIV (*n* = 1), long‐term prednisone (*n* = 1), antirejection medications following solid organ transplantation (*n* = 5), and anti‐TNF‐alpha antibody therapy (*n* = 1). Median follow‐up for all 62 cases who received first‐line chemotherapy was 775 days (range 201–2056 days). Median follow‐up at the end of the study period among the 14 living patients who received first‐line chemotherapy was 894 days (range 201–1554 days). Median follow‐up at the end of the study period among all 30 patients who received second‐line chemotherapy was 634 days (range 201–2056 days) and among the four living patients who received second‐line chemotherapy was 365.5 days (range 201–1477 days). Median time from initiation of treatment to first imaging study (among 58 of the 62 patients with available radiological data) who received first‐line chemotherapy was 58 days (range 10–274 days).

**Table 1:** Demographics of study cohort

Clinical characteristics	First line	%	Second line	%
Number of patients	62	100	30	100
Median age (range)	68.4 (46–96)		69.7 (49–96)	
Sex				
Male	47	76	24	80
Female	15	24	6	20
Patient categories				
No immune suppression	48	77	26	83
Systemic immune suppression	14	23	4	17
Immunosuppression type				
Disease‐associated immune suppression (CLL, NHL, MF, HIV)	7	11	3	10
Iatrogenic immune suppression including long‐term prednisone, anti‐TNF‐alpha, antirejection medications for solid organ transplant recipients	7	11	1	3
Stage^a^ at the time of MCC diagnosis				
IA	8	12	2	7
IB	3	5	—	0
IIA	6	10	3	10
IIB	4	6	3	10
IIC	1	2	—	0
IIIA	19	31	7	23
IIIB	12	19	6	20
IV	9	15	9	30
Vital status at the end of study period				
Alive	14	23	4	13
Dead	48	77	26	87
Cause of death				
MCC	47	98	25	96
Non‐MCC	0	0	0	0
Unknown^b^	1	2	1	4

MCC, Merkel cell carcinoma; CLL, chronic lymphocytic leukemia; NHL, non‐Hodgkin lymphoma; MF, mycosis fungoides; HIV, human immunodeficiency virus.

a AJCC 7th Edition Stage.

b First‐line patient was known to have large Merkel cell carcinoma (MCC) burden at the time of death.

### First‐line chemotherapy

**Figure 1:**
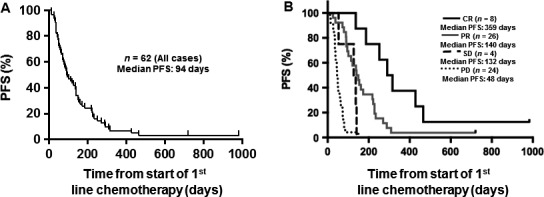
Response to first‐line chemotherapy. (A) Progression‐free survival (PFS) among all 62 patients with distant metastatic disease who received first‐line chemotherapy. (B) PFS is depicted among the patients based on their initial responses to first‐line chemotherapy.

**Figure 2:**
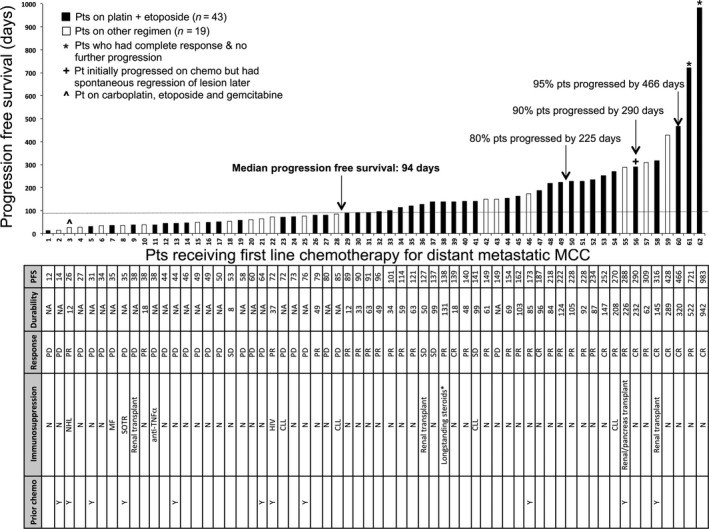
Progression‐free survival in MCC patients following first‐line chemotherapy for distant metastatic disease. Table shows the immune status of each patient and their respective response to first‐line regimen. Platin plus etoposide was the most common first‐line chemotherapy in the study population. “Prior chemo” indicates patient had received previous adjuvant chemotherapy.

Although there were 17 distinct first‐line chemotherapeutic regimens represented in this cohort, 69% (43/62) of patients received etoposide together with either carboplatin (*n* = 31) or cisplatin (*n* = 12) (Table**** 2). The initial response rate for first‐line chemotherapy was 55% (34/62 patients; 8 CR and 26 PR). The median time between start of chemotherapy and initial documentation of response was 49 days (range 7–121 days). Of the 28 (45%) patients who did not achieve an objective response, 24 patients had progressive disease and four had stable disease. Median PFS among all 62 patients who received first‐line chemotherapy was 94 days (range 12–983 days) (Fig.**** 1A). Among the eight patients who had a CR, median PFS was 303 days (range 139–983 days) and among the 26 patients with a PR, median PFS was 145 days (range 26–721 days) (Fig.**** 1B). Notably, among all 62 patients who received first‐line chemotherapy, 80% developed progressive disease by 225 days and 95% by 466 days (Fig.**** 2). The median DOR (for patients with CR and PR) for first‐line chemotherapy was 85 days (range 12–942 days) after best response was noted. The median DOR for CR was 190 days (range: 18–942 days) and for PR 63 days (range: 12–666 days).

**Table 2:** Regimen and response data for first‐ and second‐line chemotherapy

	CR	PR	SD	PD	Total
First‐line chemotherapy regimen					
Carboplatin + VP‐16	6	13	3	9	31
Cisplatin + VP‐16	1	6		5	12
Carboplatin + Irinotecan		2			2
Cisplatin + Irinotecan		1			1
Oral VP‐16				2	2
Topotecan				2	2
Cyclophosphamide, Doxorubicin, Vincristine			1	1	2
Carboplatin				1	1
Carboplatin + Docetaxel		1			1
Carboplatin + VP‐16 + Gemcitabine		1			1
Cisplatin + CPT11				1	1
Cisplatin + VP‐16 + Topotecan				1	1
Topotecan + Vincristine		1			1
Bevacizumab + VP‐16		1			1
Paclitaxel				1	1
Adriamycin				1	1
Adriamycin + Cytoxan	1				1
Total	8	26	4	24	62
%	13	42	6	39	100
Second‐line chemotherapy regimen					
Topotecan				7	7
Paclitaxel				5	5
Cytoxan + Adriamycin + Vincristine		3		1	4
Carboplatin + Taxol				3	3
Carboplatin + VP‐16	1	1	1		3
Imatinib Mesylate				1	1
Irinotecan + Mitomycin C		1			1
Carboplatin				1	1
Cisplatin + Irinotecan				1	1
Bortezomib				1	1
Irinotecan				1	1
Thalidomide + Temozolomide		1			1
Docetaxel				1	1
Total	1	6	1	22	30
%	3.3	20	3.3	73.3	100

VP‐16, etoposide; CR, complete response; PR, partial response; SD, stable disease; PD, progressive disease.

### Second‐line chemotherapy

**Figure 3:**
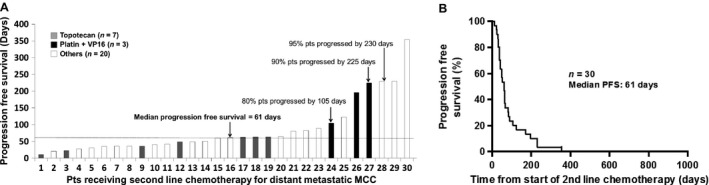
Progression‐free survival (PFS) in patients receiving second‐line chemotherapy, *n* = 30. (A) PFS among patients who received second‐line chemotherapy for distant metastatic MCC. (B) PFS from the time of initiation among patients who received second‐line chemotherapy. Median PFS was 61 days.

Thirty of the 62 patients received second‐line chemotherapy. Thirteen different chemotherapy regimens were represented among these patients. The most common agent in the second line was topotecan (7 of the 30 patients; 23%), followed by paclitaxel (5 of the 30 patients, 17%). The response rate to second‐line chemotherapy was 23% (7 of the 30 patients; 1 CR, 6 PR). Of patients, 73% (22 of the 30) had progressive disease and one (3%, 1/30) had stable disease. Median PFS among the 30 patients who received second‐line chemotherapy was 61 days (range: 11–354 days) (Fig.**** 3). Among the one patient with a CR, PFS was 105 days and among the six patients that had a PR, median PFS was 227 days (range: 60–354 days). Of patients treated with second‐line chemotherapy, 80% developed progressive disease by 105 days, while 95% did so by 230 days. For patients who had a CR or PR following second‐line chemotherapy, the median DOR was 101 days after best response (range: 6–225 days) (Table**** 3). For the one patient who had a CR, DOR was 21 days and among the six patients that had a PR, median DOR was 107 days (range: 6–225 days). Of the 30 patients who received second‐line chemotherapy, 25 died of MCC by the end of the study period, 1 patient died of an unknown cause, and 4 patients were alive (1 had a PR and 3 had PD at end of study). Of note, all seven patients who received topotecan, the most commonly used agent for second‐line chemotherapy in this cohort, experienced progressive disease without any evidence of response. The only patient to achieve complete response received carboplatin plus etoposide, although the duration of this response was only 21 days.

**Table 3:** Median progression‐free survival and durability of response for first‐ and second‐line chemotherapy regimens

	n	(%)	Median time to progression (days)	Median durability (days)
First‐line chemo response			
All cases	62	100	94	NA
CR	8	13	303	190
PR	26	42	145	63
CR + PR	34	55	168	85
CR + PR + SD	37	60	152	NA
SD	4	6	132	NA
PD	24	39	48	NA
Second‐line chemo response				
All cases	30	100	61	NA
CR	1	3	105	21
PR	6	20	227	107
CR + PR	7	23	225	101
SD	1	3	196	NA
PD	22	73	46	NA

CR, complete response; PR, partial response; SD, stable disease; PD, progressive disease; NA, not applicable.

### Overall survival

**Figure 4:**
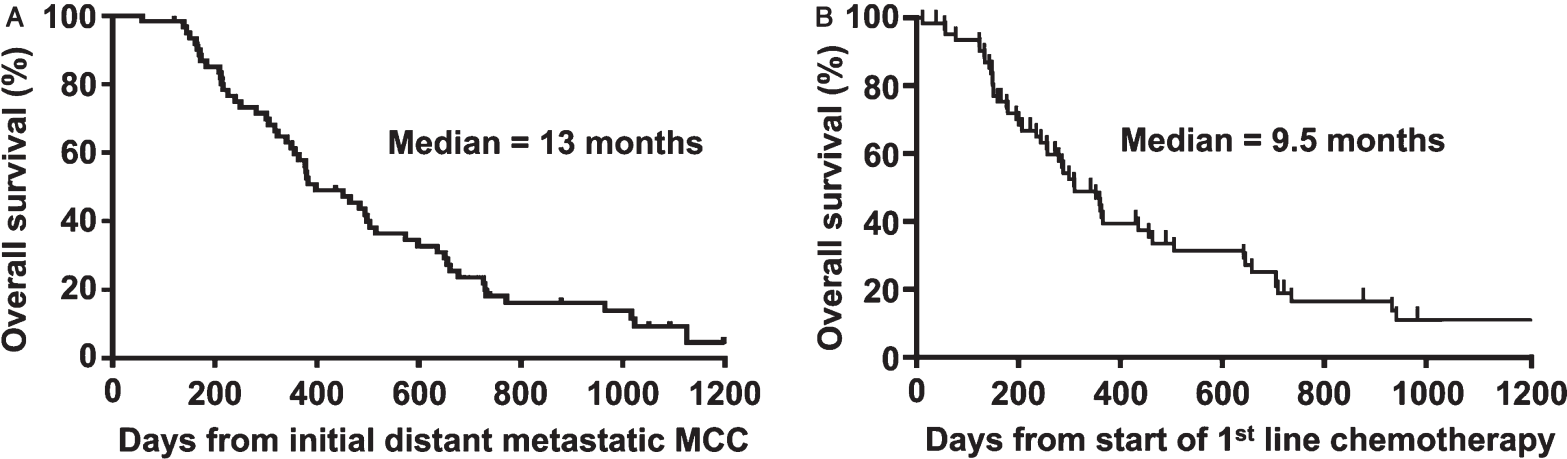
Overall survival in metastatic MCC,* n* = 62. (A) Overall survival from the time of initial diagnosis of distant metastatic disease. (B) Overall survival from the time of initiation of first‐line chemotherapy.

The median overall survival for all 62 patients from the time of their initial distant metastatic MCC diagnosis was 13 months (range: 58 days to 3.2 years) (Fig.**** 4A). Among patients receiving first‐line chemotherapy, median overall survival was 9.5 months from start of chemotherapy (Fig.**** 4B). For patients who received second‐line chemotherapy, the median overall survival time from the start of second‐line chemotherapy was 5.7 months (range: 35 days to 2.4 years; data not shown).

### Adverse events

Overall, adverse events (AEs) from chemotherapy were as expected for these cytotoxic agents in this population with a median age of 68.4 years 16. Serious AEs included febrile neutropenia (6.5%) and sepsis (4.8%). Commonly observed AEs included fatigue, alopecia, nausea, vomiting, mucositis, neutropenia–pancytopenia, and renal toxicity. No patient died due to direct toxicity from chemotherapy in this cohort.

## Discussion

The present study explored the clinical benefit of cytotoxic chemotherapy for metastatic Merkel cell carcinoma through a retrospective, detailed analysis of individual cases in a single repository. A priority for this analysis was to ensure that patients were only included if they had distant metastatic disease and were treated only with cytotoxic chemotherapy. We found that while objective responses to first‐line chemotherapy were relatively frequent (55%), durability was typically limited, with PFS of approximately 3 months among all 62 patients. Among the 30 patients who received second‐line chemotherapy, the response rate (23%) and median PFS (61 days) were lower.

For many years, cytotoxic chemotherapy has been the mainstay for the management of metastatic MCC. Two major studies have previously explored the efficacy of chemotherapy in MCC: Tai et al. (*n* = 204) and Voog et al. (*n* = 107). Importantly, however, the efficacy of chemotherapy in treating distant metastatic MCC is difficult to assess from these prior reports because they: (1) consist mostly of aggregates of individual case reports taken from the literature, and (2) include patients receiving adjuvant chemotherapy and chemoradiation in efficacy analyses. Specifically, although the reports by Voog et al. 12 and Tai et al. 11 separated metastatic disease from those with locoregional disease, it was unclear what other therapies (radiation and surgery) were given concurrently with chemotherapy making interpretation of efficacy difficult. Despite these limitations, findings from the prior studies appear quite similar to those in this study (55% initial response rate in the first line). Specifically, Voog et al. reported a 57% response rate for first‐line chemotherapy for patients with metastatic disease and Tai et al. reported a 59% response rate. The rate of response to second‐line chemotherapy was 45% as reported in Voog et al., while it was only 23% in our cohort. One possible explanation for this difference is that the Voog et al. cohort included patients with recurrent locoregional disease, whose therapy may include concurrent radiation. The response rates in such patients would be expected to be higher than for those in our cohort (comprised solely of patients with metastatic disease, excluding those who also received radiotherapy and/or surgery to the target lesions).

For patients with metastatic disease, the median overall survival from the date of chemotherapy initiation was 9 months for patients in the Voog et al. study, and it was 9.5 months in our cohort. The median PFS for all patients in our cohort from start of first‐line chemotherapy was 94 days (~3 months), with 90% of patients progressing by 290 days.

A variety of chemotherapy regimens were used in the cases from the literature and in this present cohort. In our study, a combination of etoposide plus carboplatin (or cisplatin) was the most common first‐line chemotherapy regimen (43 of the 62 cases) and had a 60% initial response rate (see Table 2). The most common regimen among the 204 cases in the Tai et al. report was cyclophosphamide/doxorubicin (or epirubicin)/vincristine ± prednisone which was used in 47 cases, with a response rate of 76% (CR 35%, PR 35%, 5% minor response). In the Voog et al.'s study, the most commonly used treatment was a “cyclophosphamide or ifosfamide‐containing regimen” used in 58 of the 107 patients, with a response rate of 64% for first‐line therapy. In patients treated with chemotherapy, a relatively high mortality rate has been reported in the literature (7.7% in Voog et al.; 3.4% in Tai et al.), however there was no mortality directly associated with chemotherapy‐related toxicity in our cohort.

There are several limitations of this study, including that the analysis was carried out retrospectively. There was no standardized regimen for first‐ or second‐line chemotherapy, in part because patients were treated at different centers. However, the diversity of therapies reflects the “real world” and allows some ability to detect whether one regimen was markedly superior to others. A further limitation is that there was no uniformity in intervals between imaging studies. Adverse event data were not uniformly available for all patients.

The present study specifically identified cases within a single repository which facilitated direct assessment of chemotherapy efficacy for metastatic MCC, including an assessment of PFS and DOR data that is not currently available in the literature. Our study confirms the previously reported high RR (>50%) with front‐line chemotherapy for metastatic MCC. Importantly, it is likely that the rate of “confirmed” responses as required by RECIST 1.1 for efficacy evaluation in prospective clinical trials (which requires that an initial response persist on a subsequent scan to register as a confirmed response) would be lower than the rate of “best responses” observed in this retrospective study as many of these initial responses were not durable until a confirmatory scan.

Because cytotoxic chemotherapy is associated with frequent response in metastatic MCC, it can be useful for palliation in symptomatic patients. Unfortunately, responses were typically not durable, and the median PFS is only 3 months. The mechanism of development of chemotherapy resistance resulting in limited durability is likely multifactorial as noted in prior studies demonstrating chemotherapy resistance via specific molecular mechanisms and changes within the tumor microenvironment 17, 18. Perhaps particularly relevant in MCC, chemotherapy suppresses the immune system. Indeed, studies have shown T‐cell lymphocyte function may remain suppressed for more than 12 months following chemotherapy administration 19, 20. Given the rarity of this cancer, logistical issues pose barriers for conducting randomized studies comparing chemotherapy with newer therapeutic modalities including immune therapies (checkpoint inhibitors, adoptive immune therapies). These data may thus be useful as a basis for comparison with the historical standard of care (cytotoxic chemotherapy) for metastatic MCC.

## Conflict of Interest

P. N. serves as a paid consultant for EMD Serono.
